# Significance of genotypes and resistance status of *Mycobacterium tuberculosis* strains in gene expression of apoptosis cell death and inflammatory pathways in A549 lung epithelial cell line

**DOI:** 10.22038/IJBMS.2024.75195.16303

**Published:** 2024

**Authors:** Rouhollah Abdolhamidi, Setareh Haghighat, Arfa Moshiri, Abolfazl Fateh, Seyed Davar Siadat

**Affiliations:** 1 Department of Microbiology, Faculty of Advanced Science and Technology, Tehran Medical Sciences, Islamic Azad University, Tehran, Iran; 2 Department of Mycobacteriology and Pulmonary Research, Pasteur Institute of Iran, Tehran, Iran; 3 Microbiology Research Center (MRC), Pasteur Institute of Iran, Tehran, Iran

**Keywords:** Alveolar epithelial cell, Drug resistance, Immune system, Mycobacterium, tuberculosis, Programmed cell death

## Abstract

**Objective(s):**

Tuberculosis (TB) has been a major health issue throughout history. As part of TB infection, host-*Mycobacterium tuberculos*is (Mtb) interactions are important. Through immune pathology and cell death control processes, Mtb infection facilitates intracellular growth. The relationship between apoptosis and inflammation in Mtb infection remains unclear. In this study, the levels of related apoptosis and inflammatory genes were assessed in A549 cells infected with a variety of Mtb strains.

**Materials and Methods:**

Mtb isolates with different phenotypes (sensitive, INH^R^, Rif^R^, MDR, and XDR) were collected from the Pasteur Institute of Iran, during this study. Whole genome sequencing was previously performed on all strains, and the Beijing genotype was selected as sensitive. Also, for other resistant strains, the New-1 genotype was available and isolated for genotype comparison. A549 lung carcinoma cells were also grown and infected with selected Mtb strains. Genes involved in inflammation and apoptosis were detected using reverse transcription-PCR (RT-PCR).

**Results:**

All sensitive strains and resistant strains were found to significantly up-regulate anti-apoptotic (*bcl2* and *rb1*), chemokine (*IL-8 *and *MCP-1*), and pro-inflammatory cytokine (*TNF-α* and *IFN-γ*) expression, while significant down-regulation was observed after 24 and 48 hr of infection in anti-inflammatory genes (IL-10) and pro-apoptotic genes (bad and bax). Besides resistance strains, Mtb genotypes also affected gene expression. The Beijing genotype (sensitive isolate) influences inflammatory and apoptotic genes more sharply than the New-1 genotype (INH^R^, Rif^R^, MDR, and XDR).

**Conclusion:**

Gene expression differences related to apoptosis and inflammation examined in the current study may be attributed to genotypes rather than resistance status since the expression of most genes has been observed to be lower in resistant strains (INH^R^, Rif^R^, MDR, and XDR belonging to the New-1 genotype) compared to sensitive strains (Beijing genotype).

## Introduction

The deadly disease of tuberculosis has long been recognized as one of the most devastating in the history of mankind (1). It is estimated that more than 1.6 million individuals have died from Tuberculosis infection. As a result, 10.6 million new TB cases have been reported (125–143 per 100,000 populations) in the latest WHO TB report. (2). In spite of much effort being spent on controlling TB, drug-resistant strains are now posing a global threat to human health (3). Multidrug-resistant tuberculosis (MDR-TB) cases worldwide rose to approximately 450,000 in 2021 (range 399,000–501,000) and 191,000 deaths (range 119,000–264,000) from MDR-TB (2). *Mycobacterium tuberculos*is (Mtb) control cannot be fully explained. It is the most common cause of death by a single infectious agent. The mechanisms for its progression cannot be fully understood. Identifying new opportunities for improving control strategies is therefore crucial. As a matter of fact, acquiring comprehensive information about TB, especially in terms of host-pathogen interactions, is crucial to controlling the disease and achieving the “END TB” goals (4). Mtb infection alters immunopathologic responses and controls cell death processes, in particular programmed cell death or apoptosis, to facilitate intracellular growth through intracellular signaling pathways (5, 6). In the immune system, apoptosis plays an essential role in preserving cellular homeostasis (7). Apoptosis is characterized by cellular membranes remaining intact, preventing leakage of cellular contents, which ensures a relatively non-inflammatory process (8). As it grows in the host cells, Mtb triggers the death of those cells so that it can escape. There are new findings that reveal the mechanisms by which this pathogen allows its bacteria to survive and avoid host immunity (9).

In the alveolar space of the lung, Mtb possesses a highly replicative and invasive phenotype as a result of interactions with epithelial cells. As part of its survival strategy in the host, Mtb also induces alveolar epithelial cells to undergo differential apoptosis and inflammatory induction (10). Induced apoptosis and inflammatory pathways are not triggered by all Mtb strains equally. There has been evidence that attenuated Mtb infection (H37Ra) induces a higher level of apoptosis than virulent Mtb infection (H37Rv). It has been suggested that virulent Mtb strains use this strategy to survive intracellularly by suppressing the immune system (11). Avirulent Mtb strains typically release cytokines and chemokines and cause apoptosis more than virulent strains (12, 13). Altering apoptosis and inflammatory factors are necessary for defense against Mtb, and in strains with less pathogenicity, the expression of these factors is greater. In the case of Mtb, however, the question of whether more resistant strains have greater pathogenicity remains unresolved (14, 15). It is common for different bacteria to increase in pathogenicity when antibiotic resistance increases, but this is not the case for Mtb (14, 16). A variety of drug-resistant strains of Mtb regulate the expression of proapoptotic and antiapoptotic genes, as well as cytokine and chemokine genes, resulting in an escalating infection by increasing virulence and decreasing immune response (17). For accurate prevention, treatment, and control of tuberculosis, it will be useful to evaluate possible links between apoptotic and inflammatory genes in host-Mtb interactions. However, there is still confusion about the relationship between apoptosis and inflammation during Mtb infection. A549 cells infected with different Mtb resistance strains (H37Rv, Sensitive, INH^R^, Rif^R^, MDR, and XDR) were used for the study to assess the levels of anti-apoptotic, pro-apoptotic, cytokine, and chemokine genes. TB severity and host susceptibility may be revealed by the presented data.

## Materials and Methods

***Mycobacterial subjects and susceptibility testing***

A total of five Mtb isolates with phenotypic characterization were obtained from the Mycobacteriology and Pulmonary Research Department of the Pasteur Institute of Iran. Several strains were included in this study, including sensitive, INH^R^, Rif^R^, MDR, XDR, and H37Rv (ATCC 27249) strains, for which the strain was used as a reference. Using the Löwenstein-Jensen (LJ) medium, drug susceptibility tests were conducted using a proportional method (18). Drug susceptibility testing had critical concentrations of 40 μg/ml (RIF), 0.2 μg/ml (INH), 4.0 μg/ml (streptomycin), 2.0 μg/ml (ethambutol), 40 μg/ml (kanamycin), 30 μg/ml (amikacin), 4.0 μg/ml (ofloxacin), and 40 μg/ml (capreomycin) for phenotypic confirmation (19). The experiments were conducted in accordance with the Centers for Disease Control and Prevention (USA) guidelines (20). 

Genotyping of all strains was previously performed using whole genome sequencing (21). The Beijing genotype was selected for this study as the sensitive isolate. Furthermore, for other resistant strains, the New-1 genotype was available and isolated for comparing genotype effects. Cultures were conducted on LJ medium for all strains. A mid-log phase (OD600: 0.6–0.9) was reached for all isolates when they were transferred into Middlebrook 7H9 broth (Sigma Aldrich, St. Louis, MO) supplemented with 0.05% Tween 80, 10% (v/v) oleic acid, albumin, dextrose, and catalase (Becton Dickinson, Oxford, UK) (22). Glass beads were used to disaggregate the clumps and strict shaking was applied. A suspension of bacteria was then placed in 15 ml plastic tubes, allowed to stand for 10 min, and then the upper half of the tubes was used for the assay. The concentration was adjusted to 1.5×10^8^ bacteria/ml using a 0.5 McFarland standard. Light microscopy was used to observe the bacteria in the suspensions stained with Ziehl-Nielsen stain. The next experiment was conducted using only dispersed inocula. A serial dilution and plating of the inocula on the LJ medium were used to determine the viable counts.

***Culture conditions and Infection with resistance strains of Mtb***

Human lung carcinoma cells A549 (ATCC CCL-185), used as AEC II, were obtained from the Mycobacteriology and Pulmonary Research Department of the Pasteur Institute of Iran. This cell line was grown in Dulbecco’s modified Eagle medium (DMEM) supplemented with streptomycin, penicillin, and 10% fetal bovine serum (FBS; Gibco, Paisley, UK) in six-well plates (Sorfa, Zhejiang, China) and incubated at 37 °C with 5% CO_2_. Before use in experiments, the cells were washed with fresh medium, seeded onto six-well plates, and allowed to grow to 60%–70% confluence. After infection with each strain (sensitive, INH^R^, Rif^R^, MDR, XDR, and H37Rv) in triplicates, A549 cells were incubated for two hours with each strain at a multiplicity of infection (MOI) of 1:1 (bacteria:cell) (23). A549 which was not infected served as the control group. After removing extracellular bacteria, infected cells were washed with DMEM and maintained in DMEM supplemented with 1% FBS and 5% CO_2_ for 24 and 48 hr. 

**Figure 1:**
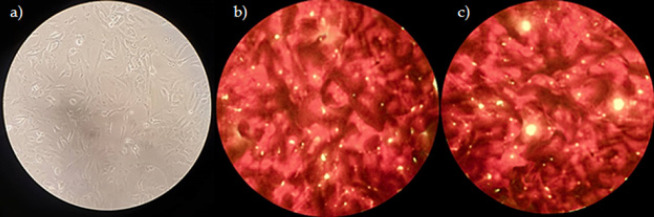
a) Uninfected A549 cells; b) Mtb-infected cells stained with auramine 24 hr after infection; c) post-infection 48-hour period

Trypan blue exclusion assay was used to assess the viability of epithelial cells infected with Mtb strains (Sigma Aldrich, Germany) according to manufacturer’s instructions (24). Infection of A549 cells with the studied Mtb strains was also performed on sterile glass coverslips. Warm DMEM was used to remove extracellular bacteria after 2 hr of infection. Infected cells remained in DMEM for 24 and 48 hr. Auramine staining was then performed to confirm bacteria internalization (Figure 1). 

***RNA extraction and cDNA synthesis***

According to the manufacturer’s instructions, total RNA was isolated from treated A549 cells using TRIzol reagent (Invitrogen, Grand Island, NY, USA). A Nanodrop spectrophotometer (ND-1000; Nanodrop Technologies) was used to determine RNA concentration and quality. A ThermoScript RT kit (Life Technologies, Rockville, MD, USA) was used to synthesize complementary DNA.

***qRT-PCR (quantitative real-time PCR)***

**Figure 2:**
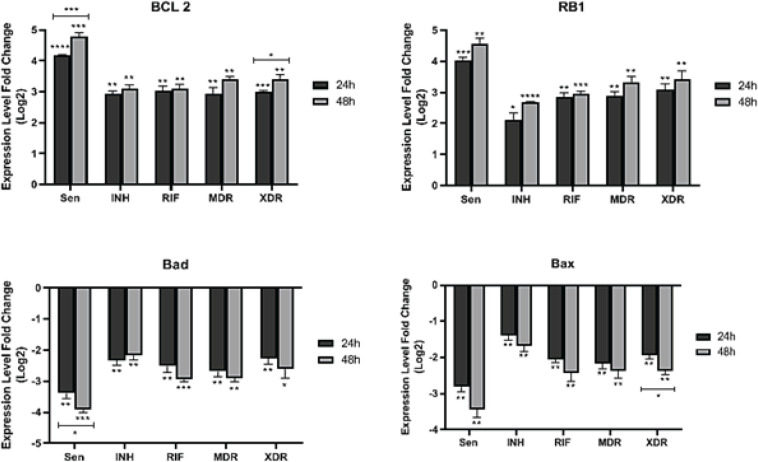
Expression level of pro-apoptotic (bad and bax) and anti-apoptotic (bcl2 and rb1) genes in post-infection time points in different resistant Mtb strains; Mann–Whitney U test

**Figure 3:**
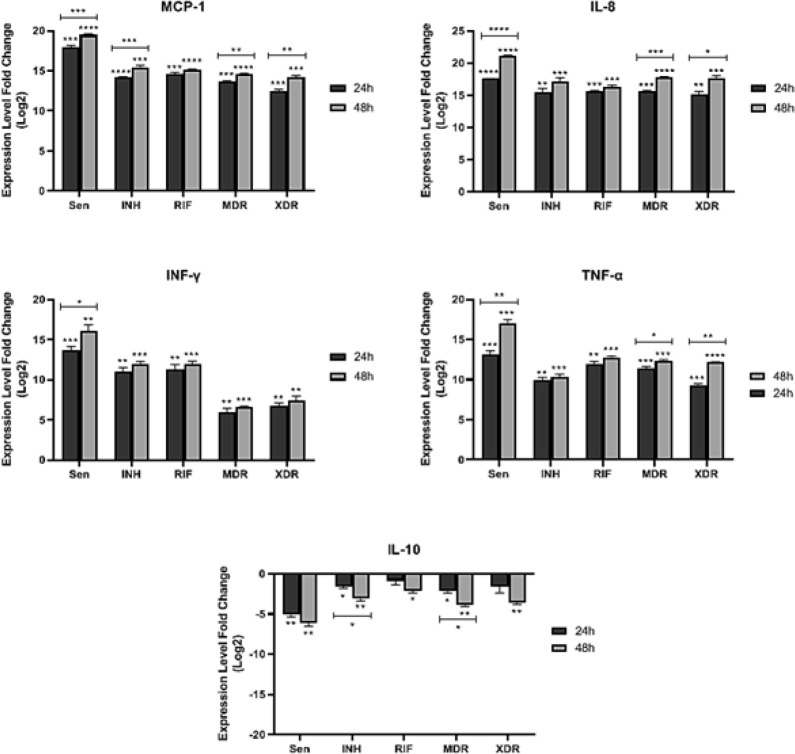
Expression level of chemokine (*MCP-1* and *IL-8*) and cytokine (*TNF-α*, I*FN-γ*, and *IL-10*) genes in time points of post-infection in different resistant strains of Mtb; Mann–Whitney U test

To detect differentially expressed pro-apoptotic (*bad* and *bax*), anti-apoptotic (*bcl2* and *rb1*), cytokine (*TNF-α*, *IFN-γ*, and *IL-10*), and chemokine (*MCP-1* and *IL-8*) genes, qRT-PCR was performed using Takara SYBR Green I kit (Takara, Dalian, China) on Roche LightCycler 2.0 System. To investigate the differential expression of apoptotic and inflammatory genes in A549 cells, the fold change of each gene in the two-time incubation period of the cells was calculated, and genes that were up and down-regulated were identified (Figures 2 & 3). A qRT-PCR reaction was carried out on 200 ng of total RNA in a final volume of 20 L qRT-PCR reaction. 95 °C for 60 sec was incubated followed by 40 cycles of 95 °C for 5 sec, 55 °C for 30 sec, and 72 °C for 30 sec, then 40 °C for 20 min. It consisted of 1 µl of product, 10 µl of SYBER Master Mix, 7 µl of desalted water, and 1 µl each of forward and reverse primers. The characteristics of the primers used in this experiment are shown in Table 1. Analyses were performed through calculation of delta CT values after normalizing each sample by its amount of *the gadh* housekeeping gene. The average of the control delta CT values in the fold changes calculation is used for the 2^–∆∆Ct^ method. A triplicate analysis of each reaction was conducted, including no-template controls.

***Statistical analysis***

For statistical analyses and graphs, GraphPad Prism 8 was used. For determining whether there was a difference between the two groups, Mann–Whitney U and Kruskal–Wallis tests were used as nonparametric tests. The significance of the study was determined by *P*-value≤0.05.

## Results

***Different expression of pro- and anti-apoptotic genes induced by different Mtb resistance strains***

Following infection by all sensitive and resistant strains, *bcl2* and *rb1* expressions were statistically significantly up-regulated, while *bad* and *bax* expressions were significantly down-regulated 24 and 48 hr post-infection (Figure 2). In addition, genotypes also affected transcription of the aforementioned pro- and anti-apoptotic genes. As compared with strains with the New-1 genotype (INH^R^, Rif^R^, MDR, and XDR), the Beijing genotype (sensitive isolate) significantly increased and decreased anti-apoptotic (*bcl2* and *rb1*) and pro-apoptotic genes (*bad* and *bax*) 24 and 48 hr post-infection. In detail, up-regulation of *bcl2 *(sensitive [*P*<0.0001; *P*=0.0007]; INH^R ^[*P*=0.001; *P*=0.001]; Rif^R ^[*P*=0.002; *P*=0.002]; MDR [*P*=0.004; *P*=0.001]; XDR [*P*=0.0001; *P*=0.001]) and* rb1 *(sensitive [*P*=0.0006; *P*=0.001]; INH^R ^[*P*=0.01; *P*<0.0001]; Rif^R ^[*P*=0.001; *P*=0.0007]; MDR [*P*=0.002; *P*=0.003]; XDR [*P*=0.003; *P*=0.005]) in infected A549 by all sensitive and resistance Mtb isolates was detected 24 and 48 hr post infection, respectively. On the other hand, down-regulation of *bad* (sensitive [*P*=0.002; *P*=0.0005]; INH^R ^[*P*=0.004; *P*=0.003]; Rif^R ^[*P*=0.006; *P*=0.0005]; MDR [*P*=0.003; *P*=0.001]; XDR [*P*=0.0075; *P*=0.01]) and *bax *(sensitive [*P*=0.002; *P*=0.004]; INH^R ^[*P*=0.009; *P*=0.009]; Rif^R ^[*P*=0.002; *P*=0.008]; MDR [*P*=0.004; *P*=0.006]; XDR [*P*=0.002; *P*=0.002]) in response to all sensitive and resistance Mtb strains was detected 24 to 48 hr after infection, respectively. 

***Expression difference of pro- and anti-apoptotic genes in time points of post-infection***

Comparison of expression difference between 24 and 48 hr post-infection showed a significant bcl2 expression difference in infected A549 by sensitive (rank of difference: -0.62; *P*=0.009) and XDR (rank of difference: -0.4; *P*=0.04) strains. Similarly, the difference in *bcl2 *expression (INH^R ^[rank of difference: -0.18; *P*=0.3]; Rif^R ^[rank of difference: -0.04; *P*=0.8]; MDR [rank of difference: -0.46; *P*=0.1]) and* rb1 *expression (sensitive [rank of difference: -0.53; *P*=0.06]; INH^R ^[rank of difference: -0.56; *P*=0.06]; Rif^R ^[rank of difference: -0.09; *P*=0.5]; MDR [rank of difference: -0.45; *P*=0.1]; XDR [rank of difference: -0.35; *P*=0.3]) has been confirmed in infected A549 by all other strains, but not significantly. In addition, infected A549 by sensitive strain (rank of difference: 0.53; *P*=0.04) showed a significant expression difference in *bad* through comparison of expression difference between 24 and 48 hr post-infection. In response to XDR strain, *bax *was significantly expressed in comparison with 24 and 48 hr after infection (rank of difference: 0.44; *P*=0.04). Expression of *bad* (INH^R ^[rank of difference: -0.16; *P*=0.4]; Rif^R ^[rank of difference: 0.43; *P*=0.1]; MDR [rank of difference: 0.21; *P*=0.3]; XDR [rank of difference: 0.33; *P*=0.3]) and *bax *(sensitive [rank of difference: 0.61; *P*=0.08]; INH^R ^[rank of difference: 0.28; *P*=0.2]; Rif^R ^[rank of difference: 0.39; *P*=0.1]; MDR [rank of difference: 0.2; *P*=0.4]) has been indicated in all other resistant strains by comparison of expression difference between 24 and 48 hr post-infection. 

***Different resistance strains of Mtb induce different levels of chemokine and cytokine expression***

There is a critical role for inflammatory pathways in the control or severity of tuberculosis. Consequently, different chemokine and cytokine genes that play a role in modulating inflammatory pathways, such as *IL-8*, *MCP-1*, *IFN-γ*, *TNF-α*, and *IL-10*, were investigated during the current study (Figure 3). In response to all sensitive and resistant strains, a statistically significant up-regulation in expression of *IL-8 *(sensitive [*P*<0.0001; *P*<0.0001]; INH^R ^[*P*=0.001; *P*=0.001]; Rif^R ^[*P*=0.0001; *P*=0.0004]; MDR [*P*=0.0001; *P*<0.0001]; XDR [*P*=0.001; *P*=0.0007]), *MCP-1 *(sensitive [*P*=0.0002; *P*<0.0001]; INH^R ^[*P*<0.0001; *P*=0.0002]; Rif^R ^[*P*=0.0001; *P*<0.0001]; MDR [*P*=0.0002; *P*<0.0001]; XDR [*P*=0.0005; *P*=0.0003]), *IFN-γ *(sensitive [*P*=0.0008; *P*=0.002]; INH^R ^[*P*=0.002; *P*=0.0005]; Rif^R ^[*P*=0.002; *P*=0.0008]; MDR [*P*=0.008; *P*=0.0005]; XDR [*P*=0.002; *P*=0.005]), and* TNF-α *(sensitive [*P*=0.0008; *P*=0.0006]; INH^R ^[*P*=0.001; *P*=0.0007]; Rif^R ^[*P*=0.001; *P*=0.0001]; MDR [*P*=0.0005; *P*=0.0001]; XDR [*P*=0.0007; *P*<0.0001]), respectively, 24 and 48 hr post infection of A549 cell line was detected . In contrast, *IL-10 *down-regulated in feedback to infection of the A549 cell line with sensitive [*P*=0.002; *P*=0.004]; INH^R ^[*P*=0.02; *P*=0.007]; Rif^R ^[*p*=0.2; *P*=0.01]; MDR [*P*=0.01; *P*=0.004]; XDR [*p*=0.1; *P*=0.004] Mtb strains 24 and 48 hr post-infection, respectively. Expression of these chemokine and cytokine genes in response to genotypes was also noticeable. The Beijing genotype (sensitive isolate) significantly induced up- and down-regulation of these genes 24 and 48 hr post-infection, whereas the strains with the New-1 genotype (INH^R^, Rif^R^, MDR, and XDR) did not.

***Expression difference of chemokine and cytokine genes in time points of post-infection***

Expression difference between 24 hr and 48 hr post-infection showed a significant difference in *IL-8 *chemokine in response to sensitive (rank of difference: -3.5; *P*=0.00004), MDR (rank of difference: -2.2; *P*=0.0002), and XDR (rank of difference: -2.4; *P*=0.02) strains. INH^R ^[rank of difference: -1.69; *P*=0.09] and Rif^R ^[rank of difference: -0.61; *P*=0.1] strains also showed a difference in expression of *IL-8 *chemokine between 24 and 48 hr post-infection, but not significantly. Significant difference in *MCP-1 *expression between 24 and 48 hr post-infection was detected in response to all Mtb strains (sensitive [rank of difference: -1.55; *P*=0.008]; INH^R ^[rank of difference: -1.3; *P*=0.006]; MDR [rank of difference: -0.97; *P*=0.01]; XDR [rank of difference: -1.8; *P*=0.008]) other than Rif^R ^[rank of difference: -0.54; *P*=0.05].

With regards to expression differences of anti-inflammatory (*IL-10*) and pro-inflammatory (*IFN-γ* and *TNF-α*) cytokines between 24 and 48 hr post-infection, significant differences were observed in the results in response to some sensitive and resistant strains. As presented in Figure 3, the sensitive strain showed a significant difference between 24 and 48 hr post-infection in pro-inflammatory cytokines, including *IFN-γ *(rank of difference: -2.33; *P*=0.05) and *TNF-α *(rank of difference: -3.86; *P*=0.002). In addition, a significant difference between 24 and 48 hr post-infection in the expression of *TNF-α *(MDR [rank of difference: -0.91; *P*=0.03]; XDR [rank of difference: -2.88; *P*=0.0002]) in response to most of the resistant Mtb strains has been noticed. Other resistant Mtb strains showed a difference between 24 and 48 hr post-infection in the expression of *IFN-γ* (INH^R ^[rank of difference: -1.0; *P*=0.1]; Rif^R ^[rank of difference: -0.66; *P*=0.3]; MDR [rank of difference: -0.66; *P*=0.2]; XDR [rank of difference: -0.66; *P*=0.3]) and *TNF-α *(INH^R ^[rank of difference: -0.51; *P*=0.3]; Rif^R ^[rank of difference: -0.91; *P*=0.09]). In terms of the evaluated anti-inflammatory cytokine, *IL-10*, the significant expression difference between incubation times was detected in INH^R ^(rank of difference: 1.49; *P*=0.01) and MDR (rank of difference: 1.72; *P*=0.01) strains. While other strains sensitive (rank of difference: 1.02; *P*=0.09), Rif^R ^(rank of difference: 1.22; *P*=0.1), and XDR (rank of difference: 1.98; *P*=0.07) showed a difference in *IL-10 *expression between 24 and 48 hr post-infection.

Mtb: *Mycobacterium tuberculos*is

Mtb: *Mycobacterium tuberculos*is

**Table 1:** The characteristics of the primers

	**Gene**	**Primer sequence**	**Product length**
**Cytokine ** **genes**	***TNF-α***	F- AGCCCATGTTGTAGCAAACC R- TGAGGTACAGGCCCTCTGAT	134
	***IFN-γ***	F- TCGGTAACTGACTTGAATGTCCA R- TCGCTTCCCTGTTTTAGCTGC	93
	***IL-10***	F- GCTGTCATCGATTTCTTCCC R- CTCATGGCTTTGTAGATGCCT	103
**Chemokine genes**	***IL-8***	F- TGCCAAGGAGTGCTAAAG R- CTCCACAACCCTCTGCAC	197
	***MCP-1***	F- CAGCCAGATGCAATCAATGCC R- TGGAATCCTGAACCCACTTCT	190
**Pro-apoptotic genes**	***bad***	F- CCCAGAGTTTGAGCCGAGTG R- CCCATCCCTTCGTCGTCCT	249
	***bax***	F- CCCGAGAGGTCTTTTTCCGAG R- CCAGCCCATGATGGTTCTGAT	155
**Anti-apoptotic genes**	***bcl2***	F- GGTGGGGTCATGTGTGTGG R- CGGTTCAGGTACTCAGTCATCC	89
	***rb1***	F- CTCTCGTCAGGCTTGAGTTTG R- GACATCTCATCTAGGTCAACTGC	214
**Housekeeping gene**	***gapdh***	F- GTCTCCTCTGACTTCAACAGCG R- ACCACCCTGTTGCTGTAGCCAA	131

## Discussion

An important platform for controlling Mtb infection is the study of host-Mtb interactions to identify specific host molecular signatures. By manipulating apoptosis and inflammatory pathways, epithelial cells play a critical role in initiating host-Mtb interactions. It is unclear how these two pathways interact during TB infection with different drug-resistant Mtb strains. In the apoptotic pathway, Bcl-2 proteins play a crucial role as intracellular checkpoints (25). It consists of members that are pro- and anti-apoptotic and are involved in inflammatory responses. Several death stimuli, including cytokine withdrawal, affect the inflammatory level of inflammatory responses when pro- and anti-apoptotic members are expressed (26). According to data related to TB infection, bacterial agents induce host cell apoptosis by activating specific components of apoptotic pathways, modulating intracellular mycobacteria killing, and triggering adaptive immunity (27). There may, however, be a variety of status, depending on the genotype or resistance strain involved (28, 29). In order to understand TB pathogenesis and effectively control TB, it may be useful to understand host-Mtb interactions (30). This study aimed to determine the expression levels of pro-apoptotic, anti-apoptotic, cytokine, and chemokine genes in infected alveolar epithelial cells (A549) infected with resistant Mtb strains (H37Rv, Sensitive, INHR, RifR, MDR, and XDR).

It was found that infection by all sensitive and resistant strains induced significant up-regulation in the expression of anti-apoptotic (*bcl2* and *rb1*), chemokine (*IL-8* and *MCP-1*), and pro-inflammatory cytokine (*IFN-γ* and* TNF-α*) genes, whereas significant down-regulation was observed in pro-apoptotic (*bad* and *bax*) and anti-inflammatory (*IL-10*) genes 24 and 48 hr post-infection. Aside from resistance strains, Mtb genotypes also affected the expression of the aforementioned genes. In comparison with strains with the New-1 genotype (INH^R^, Rif^R^, MDR, and XDR), the Beijing genotype (sensitive isolate) induced up- and down-regulation of the mentioned inflammatory and apoptotic genes more sharply. In accordance with some evidence, the Beijing strains are associated with a stronger virulence capability and a hyperinflammatory response (31). Mtb strains with a low ability to cause disease, such as avirulent strains, stimulate host cells to undergo apoptosis, resulting in an impermeable envelope that prevents bacteria from spreading (32). The process results in the containment and killing of bacteria as well as the rapid priming of immune cells. Contrary to this, virulent mycobacteria strains, such as the Beijing genotype, fail to undergo apoptosis and kill host cells by necrosis, resulting in a permeable cell membrane that enables bacteria to escape and spread (13, 32, 33). The main factors that contribute to the lethal ‘success’ of this pathogen are its ability to manipulate the inner environment of cells, especially alveolar epithelial cells. These cells serve as a favored site for intracellular survival and growth (32). It is believed that epithelial cells are the most important producers of chemokines and cytokines during TB infection, which induce local inflammatory responses and also influence apoptosis pathways (34, 35). During TB infection, highly secreted *IL-8* promotes inflammation by overexpressing bad and bax genes in host cells (36). Apoptosis is also regulated by *MCP-1* through the monocyte chemotactic protein-1-induced Ca2+-sensing receptor (CaSR) and protein-1 (MCPIP1) (37). In terms of cytokines, TNF-α increased caspase activity, and induced pro-apoptotic genes (*bad* and *bax*) (38). 

It has been shown that A549 cells express pro-apoptotic genes (*bad*, *bax*, and *fas*) and undergo apoptosis in the presence of IFN-γ and activation of the apoptotic pathways (39). Through a STAT3-dependent mechanism, IL-10 also assists in the induction of apoptosis in cells (40). Altogether, cytokine and chemokine genes are essential for primary defense against Mtb. It has been shown that increased cytokine and chemokine gene expression is usually seen in less pathogenic strains (12). It should be noted, however, that the issue of whether or not more resistant Mtb strains are pathogenic is still being debated in the case of this pathogen (14). There is no question that certain genotypes of Mtb, in particular the Beijing genotype, are identified as showing potentially higher pathogenicity than others (41). Even though various factors contribute to a balance between the survival of Mtb inside the cell, its reproduction, and transmission, there is still very little understanding of how pathogenic mechanisms work. As a whole, the ability of different strains of Mtb to maintain this equilibrium within the cell (survival, intracellular reproduction, and transmission) is used to determine their pathogenic potential (14). There is also a claim that the ability of MTB strains with antibiotic resistance to induce this equilibrium state determines their pathogenicity as well. The action of antibiotics is closely related to the targeting of key points of organism survival. These include the synthesis of cell walls, the regulation of chromosome form, the transcription of RNA, and the translation of proteins (42). Because most of the mechanisms behind antibiotic resistance are related to a reduction in Mtb’s growth and proliferation as well as an increase in division time, it is likely that some strains resistant to antibiotics may not have the ability to maintain a state of biological equilibrium due to their inability to maintain this state (43). Essentially, or to put it another way, different Mtb strains may have different growth rates and pathogenicity characteristics regardless of whether or not they possess antibiotic resistance patterns (14). Despite this, it is important to keep in mind that in some resistant Mtb strains, additional mutations may mean they can compensate for mutations that have occurred and maintain this balance, thus increasing their pathogenicity (43).

## Conclusion

The difference in how genes are expressed in relation to apoptosis and inflammation examined in the current study is likely due to the genotypes of these strains rather than their resistance status, since in the case of most genes, the expression of these genes has been observed to be lower in resistant strains (INH^R^, Rif^R^, MDR, and XDR belonging to the New-1 genotype) compared to sensitive strains (belonging to the Beijing genotype). In light of this, further studies are necessary to understand the further perspectives of the association between apoptosis and inflammatory pathways, and involved mechanisms that are yet to be identified regarding their role in TB infection, as well as the possible mechanisms involved. In the long run, such studies will help to develop more efficient and effective treatments for TB, thereby reducing the global burden of this disease. Thus, to gain a deeper understanding of the TB disease and how it is transmitted, evaluating the host-Mtb interactions under different conditions of TB infection can be of significant importance.

## Authors’ Contributions

SD H and S H conceived the study; R A provided methodology; R A performed cell culture and molecular tests; SD H and A F performed validation; A M helped with statistical analysis; R A and A M performed bacterial culture; R A prepared the original draft; SD H and S H reviewed and edited the manuscript; SD H supervised; All authors have read and agreed to the published version of the manuscript.

## Conflicts of Interest

The authors declare that no conflict of interest exists.
